# NAMPT inhibition induces ferroptosis via mitochondrial metabolic reprogramming to enhance tumour immunogenicity in glioblastoma

**DOI:** 10.3389/fimmu.2026.1721125

**Published:** 2026-03-31

**Authors:** Dongxu Zhao, Lin Duan, Hang Ren, Hang Zhang, Menghui Zhang, Zhuohang Liu, Hailan Wang, Wenxuan Lu, Wenhan Shi, Qingwang Hou, Tianxiao Li, Ming Li

**Affiliations:** 1 Department of Cerebrovascular Disease and Neurosurgery, Henan University People’s Hospital, Henan Provincial People’s Hospital, Zhengzhou, China; 2 Henan Provincial People’s Hospital, Department of Cerebrovascular Disease and Neurosurgery, Zhengzhou University People’s Hospital, Zhengzhou, China

**Keywords:** ferroptosis, glioblastoma, immunogenicity, mitochondrial metabolic reprogramming, NAMPT inhibitor

## Abstract

**Background:**

Ferroptosis has recently been recognised as an immunogenic form of regulated cell death. Nicotinamide phosphoribosyltransferase (NAMPT), a key enzyme in the NAD^+^ salvage pathway, is highly expressed in glioblastoma and represents a promising metabolic target.

**Methods:**

We investigated the effects of the NAMPT inhibitor GMX1778 on ferroptosis and tumour immunogenicity in glioblastoma cells. Mitochondrial NAD^+^ levels, SIRT3 activity, and ferroptosis-related markers were analysed by biochemical assays and western blotting. The PERK–CHOP pathway and markers of enhanced immunogenicity (calreticulin exposure, ATP release, HMGB1 release) were evaluated. The role of ferroptosis was verified using inducers and inhibitors. Macrophage polarisation was assessed in co-culture with treated tumour cells. *In vivo* immune responses were examined using a prophylactic vaccination model in GL261 glioma-bearing mice.

**Results:**

NAMPT inhibition by GMX1778 depleted mitochondrial NAD^+^, suppressed SIRT3, and induced ROS accumulation with downregulation of GPX4, leading to ferroptosis. This was accompanied by strong activation of ER stress and increased markers of immunogenicity. Ferroptosis inducers amplified, while inhibitors suppressed, these responses. GMX1778-treated glioma cells promoted macrophage polarisation from an M2 to M1 phenotype. *In vivo*, GMX1778 pre-treatment significantly reduced intracranial tumour incidence and enhanced anti-tumour immune responses in mice.

**Conclusion:**

NAMPT inhibition induces ferroptosis via mitochondrial metabolic reprogramming and subsequently enhances tumour immunogenicity through ER stress activation. These findings highlight a novel metabolic–immune mechanism and suggest that NAMPT inhibitors could serve as promising agents for glioblastoma therapy.

## Introduction

Glioblastoma (GBM) is amongst the most aggressive and lethal tumours of the central nervous system (1). Although temozolomide (TMZ) remains the standard first-line chemotherapeutic agent, its efficacy is often compromised by the development of tumour cell resistance (2, 3). In recent years, metabolic reprogramming has emerged as a hallmark of cancer, offering new avenues for therapeutic intervention. GBM exhibits a marked dependence on the nicotinamide adenine dinucleotide (NAD^+^) salvage pathway, in which nicotinamide phosphoribosyltransferase (NAMPT), a rate-limiting enzyme, is highly expressed (4). Inhibition of NAMPT depletes intracellular NAD^+^ levels (5–7), thereby impairing tumour metabolic activity and potentially enhancing anti-tumour immune responses through modulation of the tumour microenvironment. Notably, most existing studies have focused on the cytosolic NAD^+^-dependent deacetylase SIRT1 (4, 8), whereas the role of the mitochondrial deacetylase SIRT3, also regulated by NAMPT, remains largely unexplored in the context of tumour biology.

Ferroptosis is a newly recognised form of programmed cell death characterised primarily by iron-dependent lipid peroxidation and excessive accumulation of ROS (6). It exerts direct cytotoxic effects on tumour cells by damaging membrane lipids, particularly through the build-up of lipid peroxides within mitochondrial membranes, thereby exacerbating mitochondrial dysfunction (9). NAMPT inhibitors may influence mitochondrial metabolism by depleting NAD^+^, a critical co-factor for mitochondrial enzymes (4, 10, 11). Notably, NAD^+^ serves as a key regulator of SIRT3, a mitochondrial NAD^+^-dependent deacetylase involved in maintaining redox homeostasis and lipid metabolism (12, 13). Therefore, NAMPT inhibition may disrupt SIRT3 activity, leading to ROS accumulation and lipid peroxidation, ultimately triggering ferroptosis. This mechanism offers a novel therapeutic perspective for the treatment of GBM.

In addition to directly inducing tumour cell death, ferroptosis has been shown to influence the tumour immune microenvironment. Our previous studies demonstrated that the NAMPT inhibitor GMX1778 significantly increases the infiltration of CD3^+^, CD4^+^ and CD8^+^ T cells, while concurrently reducing the population of M2 macrophages in a murine glioblastoma model (14). This reshaping of the immune cell composition suggests that NAMPT inhibitors not only exert direct cytotoxic effects on tumour cells via metabolic disruption but also enhance tumour immunogenicity. In particular, the accumulation of ROS and activation of endoplasmic reticulum (ER) stress during ferroptosis can act as potent danger signals, leading to the release of immunogenic molecules such as calreticulin (CRT), ATP and HMGB1 (15–17). These events promote macrophage polarisation towards the pro-inflammatory M1 phenotype and facilitate the recruitment and activation of effector T cells, thereby enhancing anti-tumour immune responses.

Accordingly, this study aimed to systematically elucidate the mechanism by which NAMPT inhibition induces ferroptosis and enhances tumour immunogenicity through SIRT3-mediated mitochondrial metabolic reprogramming. By investigating the crosstalk between ferroptosis and immune activation, we seek to provide a theoretical foundation and experimental evidence for the combined application of metabolic-targeted therapy and immunotherapy in the treatment of GBM.

## Materials and methods

### Public databases and RNA sequencing analysis

Transcriptomic data from patients with glioma were obtained from The Cancer Genome Atlas (TCGA) and the Chinese Glioma Genome Atlas (CGGA) databases and integrated for analysis. These datasets were used to evaluate the relationship between the expression profiles of key genes and patient prognosis.

Additionally, mRNA sequencing was performed on two groups of U251 glioma cells: a control group (n = 3) and a GMX1778-treated group (n = 3). Detailed data are provided in the **Supplementary Materials** (**Data S1**). Total RNA was extracted using the MJzol Animal RNA Isolation Kit (Majorbio) following the manufacturer’s protocol. The extracted RNA was subsequently purified with the RNAClean XP Kit (Beckman Coulter) and RNase-Free DNase Set (QIAGEN). Purified RNA samples underwent poly(A) mRNA enrichment, fragmentation and first- and second-strand cDNA syntheses. These steps were followed by end repair, 3′ adenylation, adaptor ligation and PCR amplification to construct the sequencing libraries. Library concentrations were quantified using a Qubit 2.0 fluorometer, and fragment size distribution was assessed using the Agilent 4200 TapeStation system. Sequencing was conducted on the Illumina NovaSeq 6000 platform using paired-end 150 bp (PE150) reads.

### Cell culture

The cell lines used in this study included the human glioma cell line U251, the murine glioma cell line GL261, the human monocytic cell line THP-1 and the murine macrophage cell line RAW264.7. All cell lines were obtained from the Cell Bank of the Shanghai Institute of Biological Sciences (Shanghai, China).

Adherent cells were cultured in high-glucose Dulbecco’s Modified Eagle Medium (Biological Industries, BI) supplemented with 10% foetal bovine serum (FBS; BI) and 1% penicillin–streptomycin (Meilunbio) and maintained at 37 °C in a humidified atmosphere containing 5% CO_2_. Suspension cells were cultured in RPMI-1640 medium (BI) supplemented with 10% FBS (Gibco) and 1% penicillin–streptomycin under the same conditions.

### Cell viability assay

Cell viability was assessed using the Cell Counting Kit-8 (CCK-8; Meilunbio). U251 and GL261 cells were seeded in 96-well plates at a density of 2,000 cells per well. Cells were treated with varying concentrations of the NAMPT inhibitor GMX1778 (0, 5, 10, 20, 40, 80, 160, 320, 640 and 1280 nM) for 24, 48 and 72 h. After each treatment period, CCK-8 reagent was added to each well, followed by incubation at 37 °C for 2 h. Absorbance was measured at 450 nm using a microplate reader to quantify cell viability. Through the results of dose–response analysis, 48 h of treatment with GMX1778 at concentrations of 5 and 10 nM for U251 cells and 50 and 100 nM for GL261 cells was selected for subsequent experiments. Detailed results are presented in **Supplementary Figure 1**.

### Western blot

Protein expression levels were assessed by Western blot. Treated U251 and GL261 cells were lysed using RIPA lysis buffer, and protein concentrations were quantified using the BCA Protein Assay Kit (Solarbio, PC0020). Equal amounts of protein samples were separated by SDS-PAGE and transferred onto nitrocellulose membranes (HATF00010, Sigma). After blocking, membranes were incubated with primary antibodies overnight at 4 °C, followed by incubation with secondary antibodies at room temperature for 1 h after washing with TBST.

Protein bands were visualised using enhanced chemiluminescence reagents (P10300, NcmBiotech), and band intensities were quantified using ImageJ software. All antibodies were purchased from Proteintech Group (Wuhan, China).

### Plasmid construction and transfection

All plasmids used in this study were purchased from Vigenebio. SIRT3 knockdown was achieved using an shRNA-based approach, and three shRNA vectors targeting human Sirt3 were custom synthesised by Vigenebio (order no. 20260128009) with the following sequences: shRNA1, GTGGGTGCTTCAAGTGTTGTTCTCGAGAACAACACTTGAAGCACCCACTTTTTT; shRNA2, CCCAACGTCACTCACTACTTTCTCGAGAAAGTAGTGAGTGACGTTGGGTTTTTT; shRNA3, GCGGCTCTACACGCAGAACATTTCAAGAGAATGTTCTGCGTGTAGAGCCGCTTTTTT. Cell transfection was performed according to the manufacturer’s instructions for the transfection reagent, and cells were harvested at the indicated time points for subsequent protein analyses and functional assays. The efficiency of SIRT3 overexpression or knockdown was validated by western blotting.

### Immunofluorescence assay

Immunofluorescence was performed to evaluate the expression, subcellular localisation and marker changes of key intracellular proteins. U251 and GL261 cells were seeded on glass coverslips and treated with the indicated compounds. After treatment, cells were fixed with 4% paraformaldehyde for 15 min and permeabilised with 0.3% Triton X-100 for 10 min at room temperature. Non-specific binding was blocked with 5% bovine serum albumin for 1 h. Cells were then incubated with primary antibodies overnight at 4 °C, followed by incubation with fluorescence-conjugated secondary antibodies for 1 h at room temperature. Nuclei were stained with DAPI (Meilunbio), and coverslips were washed with PBS and mounted for imaging. Intracellular ROS and mitochondrial superoxide levels were assessed using ROS-specific probes, MitoSOX™ Deep Red and MitoBright LT Green (Dojindo, Japan). All images were acquired and analysed by using a Leica SP8 laser scanning confocal microscope.

### Flow cytometry

Single-cell suspensions were prepared from co-cultured THP-1 and RAW264.7 cells. Cells were initially incubated with Fc Block (BioLegend, 422302) for 10 min at 4 °C to prevent nonspecific antibody binding. Subsequently, cells were stained with fluorophore-conjugated surface antibodies for 30 min at 4 °C in the dark. After staining, cells were resuspended in 1×PBS and immediately analysed using a BD LSRFortessa flow cytometer (BD Biosciences). Fluorescence data were processed and analysed using FlowJo software.

### Immune cell co-culture

THP-1 cells were differentiated into M0 macrophages by treatment with 100 ng/mL phorbol 12-myristate 13-acetate (PMA) for 24 h, followed by replacement with fresh PMA-free medium; culture was continued for an additional 24 h. RAW264.7 cells were cultured directly and used at the logarithmic growth phase.

U251 and GL261 glioma cells were seeded into 6-well plates and treated with the NAMPT inhibitor GMX1778 at specified concentrations (U251: 5 and 10 nM; GL261: 50 and 100 nM) for 48 h. The supernatants from treated glioma cells were collected and used as conditioned medium for macrophage culture.

Conditioned media were applied to THP-1-derived macrophages and RAW264.7 cells along with 20 ng/mL IL-4, and the cells were incubated for 48 h. The expression of CD86 and CD206 was then assessed by immunofluorescence and flow cytometry to evaluate the effects of GMX1778-induced glioma-derived factors on macrophage polarisation. A schematic of the co-culture setup is provided in **Supplementary Figure 2**.

### Prophylactic vaccination model

C57BL/6 mice were housed under temperature-controlled conditions with a 12-hour light/dark cycle and provided ad libitum access to food and water. A total of 20 mice were randomly divided into two groups: a PBS control group and a GMX1778 treatment group. Each mouse was subcutaneously vaccinated on the left flank with 5 × 10^4^ GL261-Luc cells pre-treated with the NAMPT inhibitor GMX1778 or with PBS alone as a control. After 7 days, 5 × 10^4^ untreated wild-type GL261-Luc cells suspended in 5 μL of PBS were stereotactically implanted into the right frontal lobe of the brain. Tumour incidence was monitored on days 7 and 10 after intracranial implantation by using an *in vivo* imaging system (IVIS Spectrum, PerkinElmer, USA). All animal procedures were performed in accordance with institutional guidelines and were approved with ethical approval number SYXK-2021-0009.

### Statistical analysis

All data were analysed using GraphPad Prism version 9.0. Comparisons between two groups were performed using unpaired Student’s t-tests. For comparisons involving three or more groups, one-way ANOVA was applied. Tumour volume data were analysed using two-way ANOVA, followed by Huynh–Feldt correction and Tukey’s *post hoc* test. Survival data were presented as Kaplan–Meier survival curves, and statistical differences were assessed using the log-rank (Mantel–Cox) test. A P value < 0.05 was considered statistically significant. Statistical significance in figures is indicated as follows: P < 0.05 (*), P < 0.01 (**), P < 0.001 (***) and P < 0.0001 (****); ‘ns’ indicates no statistically significant difference.

## Results

### High expression of NAMPT in GBM and its inhibitor-induced metabolic reprogramming

**Figure 1:**
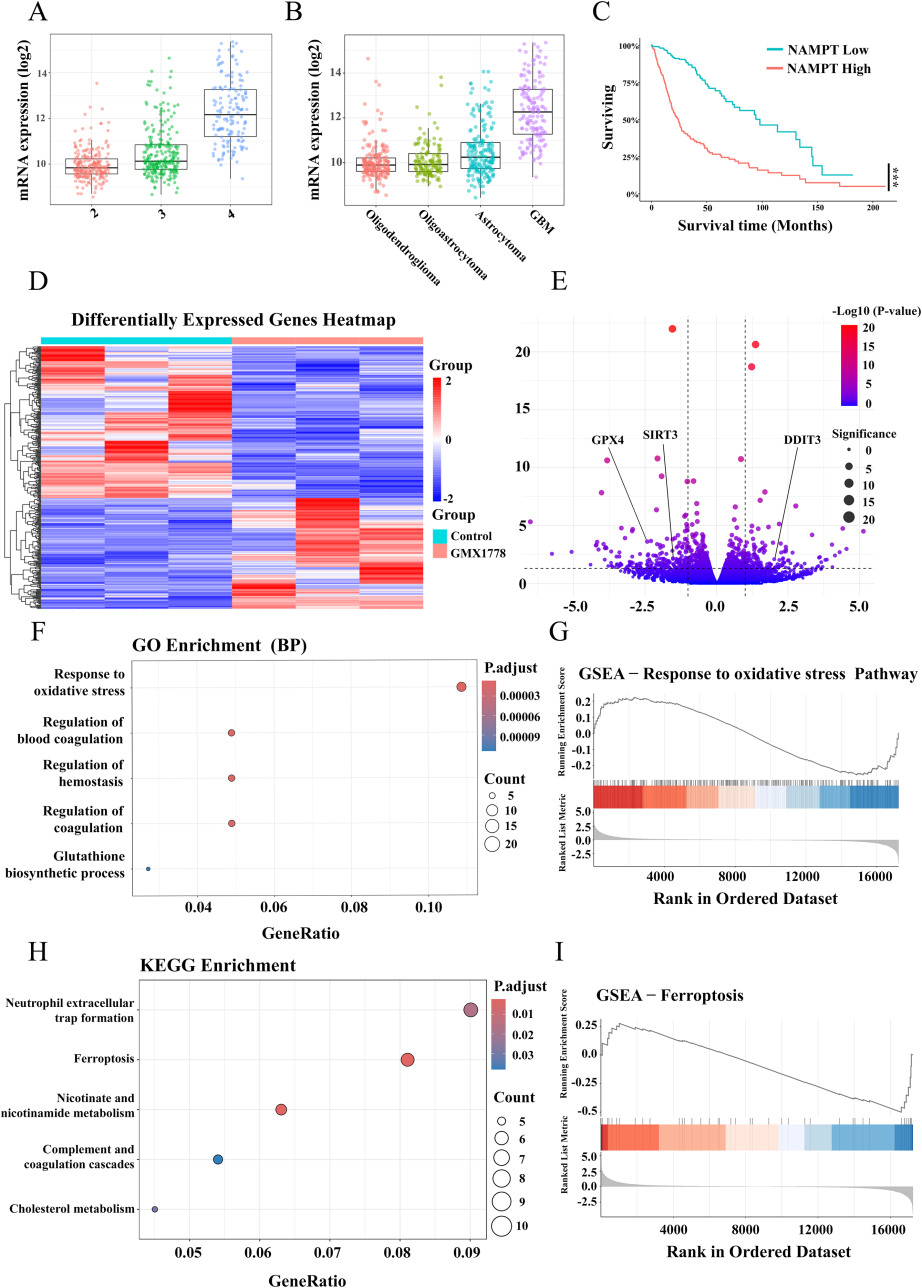
NAMPT expression in glioblastoma and the transcriptional impact of NAMPT inhibition. **(A)** Box plot showing NAMPT mRNA expression across glioma grades (WHO grade 2, 3, and 4). **(B)** Box plot comparing NAMPT mRNA expression among different glioma subtypes (oligodendroglioma, astrocytoma, and glioblastoma. **(C)** Kaplan–Meier survival analysis comparing overall survival between patients with high and low NAMPT expression. **(D)** Heatmap displaying DEGs in U251 cells treated with the NAMPT inhibitor GMX1778. **(E)** Volcano plot highlighting DEGs, with key ferroptosis-related genes. **(F)** GO enrichment analysis showing the top five enriched BPs among DEGs, including oxidative stress response, regulation of blood coagulation, and glutathione biosynthesis. **(G)** GSEA indicating significant enrichment of the oxidative stress pathway in GMX1778-treated cells. **(H)** KEGG pathway enrichment analysis identifying the top five pathways enriched among DEGs, including ferroptosis, neutrophil extracellular trap formation, and cholesterol metabolism. **(I)** GSEA confirming significant enrichment of the ferroptosis pathway in the GMX1778 treatment group.Data represent at least three independent experiments. Results are presented as mean ± standard deviation (SD). no statistically significant difference(ns), P < 0.001 (***).

Analysis of the TCGA database revealed a significant differential expression of NAMPT in gliomas, with mRNA levels increasing progressively with tumour grade. In particular, NAMPT expression was significantly higher in grade 4 gliomas than in grade 2 and grade 3 gliomas (**Figure 1A**). Further analysis across histological subtypes showed that NAMPT expression was highest in GBM, with significantly elevated levels compared with oligodendrogliomas and astrocytomas (**Figure 1B**). Kaplan–Meier survival analysis indicated that patients with high NAMPT expression had significantly shorter overall survival than those with low NAMPT expression, suggesting a strong association between elevated NAMPT levels and poor prognosis (**Figure 1C**). These findings were independently validated using the CGGA dataset (**Supplementary Figure 3**).

Given that NAD^+^ biosynthesis in malignant tumours primarily relies on the salvage pathway, NAMPT represents a potential metabolic vulnerability in GBM. To investigate this, we performed RNA sequencing on U251 cells treated with the NAMPT inhibitor GMX1778. The results showed a remarkable change in the transcriptomic profile upon treatment. A heatmap of differentially expressed genes revealed widespread expression changes between the GMX1778-treated and control groups (**Figure 1D**). These changes were further illustrated in a volcano plot (**Figure 1E**), highlighting significant alterations in key ferroptosis-related genes, including GPX4, SIRT3 and DDIT3.

Gene ontology (GO) enrichment analysis demonstrated that differentially expressed genes were significantly enriched in biological processes associated with ferroptosis, such as oxidative stress response and glutathione metabolism (**Figure 1F**). Gene Set Enrichment Analysis (GSEA) further confirmed the enrichment of oxidative stress-related pathways (**Figure 1G**). Kyoto Encyclopaedia of Genes and Genomes (KEGG) pathway analysis revealed that these genes were primarily involved in ferroptosis, nicotinamide metabolism and cholesterol metabolism (**Figure 1H**), with GSEA again validating the significant enrichment of ferroptosis-related pathways (**Figure 1I**). Together, these results indicated that NAMPT inhibition significantly induced ferroptosis in GBM by modulating the expression of ferroptosis-associated genes, suggesting a potential metabolic strategy for targeting glioblastoma.

### NAMPT inhibition induces ferroptosis through SIRT3 regulation

**Figure 2:**
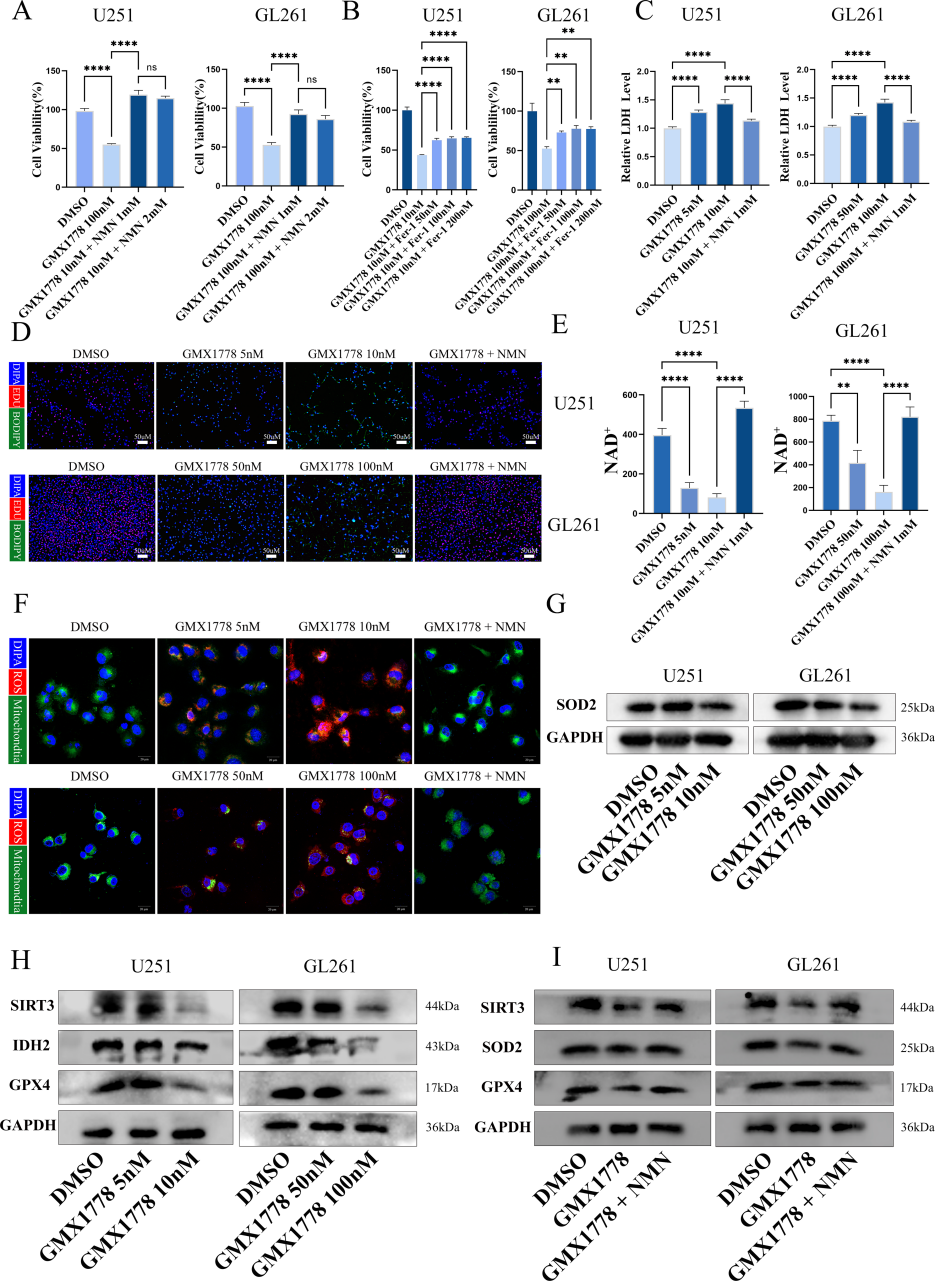
NAMPT Inhibitor GMX1778 Induces ROS Accumulation and Mitochondrial NAD^+^ Depletion. **(A, B)** Cell viability assays showing that GMX1778 significantly reduced the viability of U251 and GL261 cells; co-treatment with Fer-1 or NMN partially reversed this effect. **(C)** LDH release assay measuring cytotoxicity in U251 and GL261 cells following GMX1778 treatment. **(D)** Fluorescence microscopy analysis of intracellular lipid droplets using BODIPY 493/503 (green) and proliferative activity using EDU (red). **(E)** Quantification of mitochondrial NAD^+^ levels in U251 and GL261 cells after treatment with GMX1778 and/or NMN. **(F)** Confocal fluorescence microscopy detecting mitochondrial ROS levels and mitochondrial distribution. **(G–I)** Western blot analysis of ferroptosis-related proteins including SIRT3, IDH2, SOD2, and GPX4.Data are representative of at least three independent experiments. Results are presented as mean ± standard deviation (SD). no statistically significant difference(ns), P < 0.05 (*), P < 0.01 (**) and P < 0.0001 (****).

Bioinformatic analysis in the previous section identified NAMPT as a key metabolic vulnerability in GBM, and its inhibition was shown to induce ferroptosis. Further investigations revealed that the NAMPT inhibitor GMX1778 significantly reduced cell viability in U251 and GL261 glioma cells. This cytotoxic effect was fully reversed by supplementation with the NAD^+^ precursor nicotinamide mononucleotide (NMN; **Figure 2A**). Interestingly, partial reversal was also observed upon treatment with the ferroptosis inhibitor Ferrostatin-1 (Fer-1) (**Figure 2B**), suggesting that ferroptosis contributed to the NAD^+^-dependent cell death induced by NAMPT inhibition in GBM. To confirm the involvement of ferroptosis, we assessed the levels of lactate dehydrogenase release and lipid peroxidation. Both were significantly increased following GMX1778 treatment and were markedly reversed by NMN supplementation (**Figures 2C, D**), further supporting the ferroptotic nature of cell death.

GO analysis revealed that NAMPT inhibition induced oxidative stress, with mitochondrial ROS identified as the primary source. Experimental results demonstrated that GMX1778 substantially reduced mitochondrial NAD^+^ levels and led to pronounced mitochondrial ROS accumulation. NMN supplementation restored NAD^+^ levels and significantly reduced ROS production (**Figures 2E, F**). To elucidate the mechanism underlying mitochondrial ROS accumulation, we focused on SIRT3—a mitochondrial NAD^+^-dependent deacetylase known to regulate redox balance. GMX1778 treatment significantly suppressed SIRT3 expression, along with its downstream antioxidant targets, including isocitrate dehydrogenase 2 (IDH2) and glutathione peroxidase 4 (GPX4; **Figures 2G, H**). Expression of SOD2, another mitochondrial antioxidant enzyme, was also notably reduced. NMN treatment restored the expression of SIRT3, SOD2 and GPX4 (**Figure 2I**). Collectively, these findings demonstrated that NAMPT inhibition depleted mitochondrial NAD^+^, suppressed SIRT3 activity and disrupted the SIRT3–GPX4 antioxidant axis, leading to mitochondrial ROS accumulation and ferroptosis in GBM cells.

### NAMPT inhibition triggers ER stress via ferroptosis to enhance immunogenicity

Our previous studies demonstrated that NAMPT inhibitors enhance the immunogenicity of the tumour microenvironment. Thus, we hypothesised that this enhanced immunogenicity may be associated with ferroptosis-induced ER stress. As established in the previous section, NAMPT inhibition induces ferroptosis, characterised by excessive accumulation of intracellular ROS. Therefore, we postulated that ferroptosis-induced ROS may trigger ER stress, thereby promoting the immunogenicity of glioma cells.

**Figure 3:**
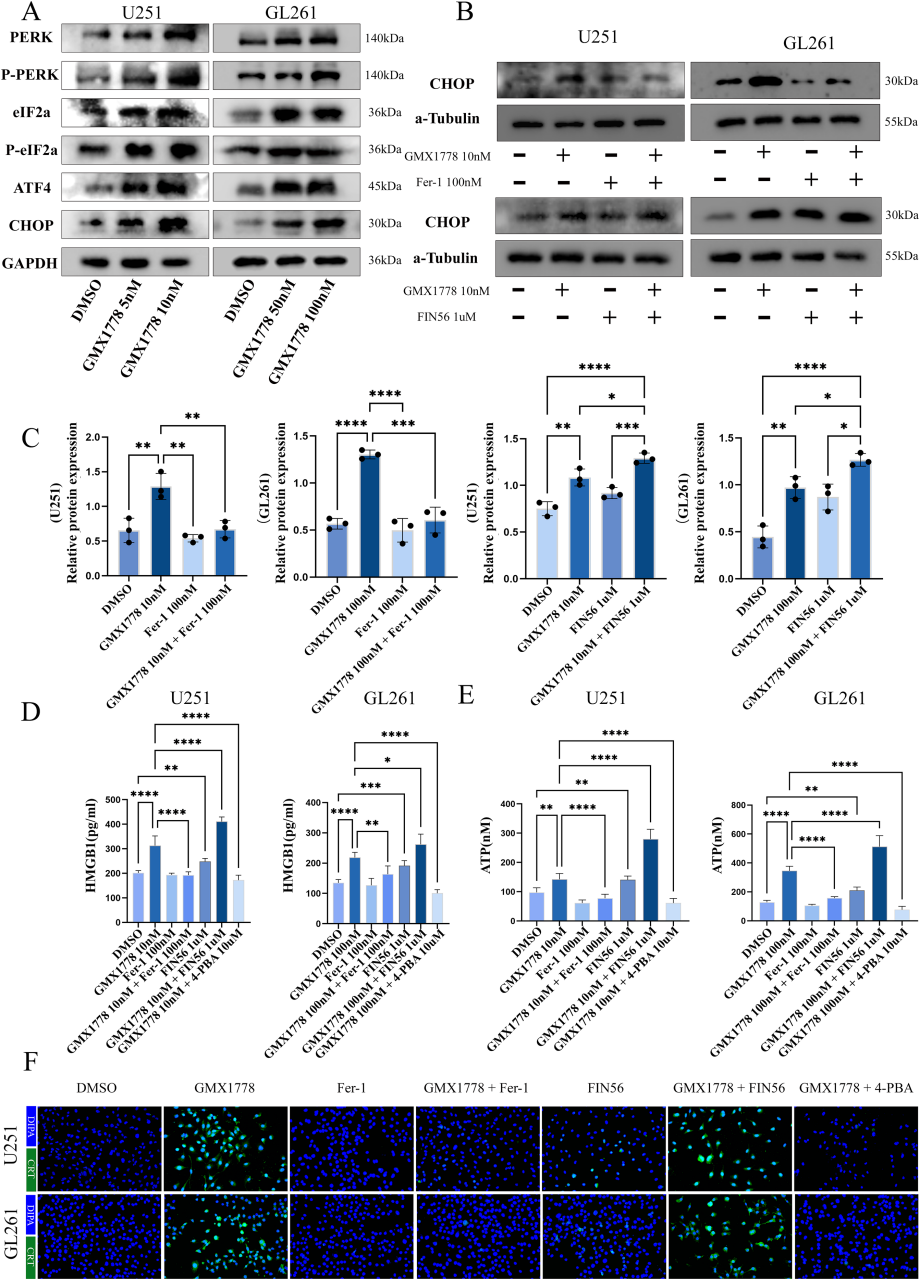
NAMPT inhibition induces endoplasmic reticulum stress and enhances tumour immunogenicity. **(A)** Western blot analysis showing increased expression of ER stress–related proteins, including PERK, phospho-PERK, eIF2α, phospho-eIF2α, ATF4, and CHOP, in U251 and GL261 cells after GMX1778 treatment. **(B, C)** Western blot detection and quantitative analysis of CHOP protein levels based on band intensity. **(D)** Enzyme-linked immunosorbent assay (ELISA) measuring HMGB1 release in culture supernatants. **(E)** Quantification of extracellular ATP release by luminescence generated through a firefly luciferase–dependent reaction. **(F)** Immunofluorescence microscopy analysis of CRT exposure on the surface of glioma cells. Data are representative of at least three independent experiments and presented as mean ± standard deviation (SD). no statistically significant difference(ns), P < 0.05 (*), P < 0.01 (**), P < 0.001 (***) and P < 0.0001 (****).

To test this hypothesis, we assessed whether NAMPT inhibition can activate ER stress. Western blot analysis revealed that GMX1778 markedly increased the expression of key proteins involved in ER stress signalling, including PERK, phosphorylated PERK (P-PERK), eIF2α, phosphorylated eIF2α (P-eIF2α), ATF4 and CHOP (**Figure 3A**), indicating that NAMPT inhibition activated the ER stress responses. To further determine whether ferroptosis mediates ER stress under NAMPT inhibition, we used the ferroptosis inhibitor Fer-1 and the ferroptosis inducer FIN56. Fer-1 treatment significantly suppressed CHOP expression, whereas FIN56 enhanced it (**Figures 3B, C**). These findings were corroborated by immunofluorescence staining of CHOP (**Supplementary Figure 4**), confirming that NAMPT inhibition activated ER stress in a ferroptosis-dependent manner.

Subsequently, we examined whether NAMPT inhibition can enhance immunogenicity by measuring classical damage-associated molecular pattern (DAMP)-related signals, including ATP release, high mobility group box 1 (HMGB1) release and calreticulin (CRT) translocation to the cell surface. GMX1778 significantly increased ATP and HMGB1 release and promoted CRT exposure (**Figures 3D–F**), indicating an enhancement of glioma cell immunogenicity. To further confirm that this immunogenicity was driven by ferroptosis-mediated ER stress, we employed Fer-1, FIN56 and the ER stress inhibitor 4-phenylbutyric acid (4-PBA). Fer-1 and 4-PBA suppressed the expression of these immunogenicity-associated signals induced by GMX1778, whereas FIN56 enhanced their release (**Figures 3D–F**, **Supplementary Figure 5**). These results collectively demonstrated that NAMPT inhibition induced ferroptosis, which activated ER stress, ultimately leading to the enhancement of glioma cell immunogenicity.

### NAMPT inhibition enhances tumour immunogenicity via SIRT3-mediated ferroptosis

**Figure 4:**
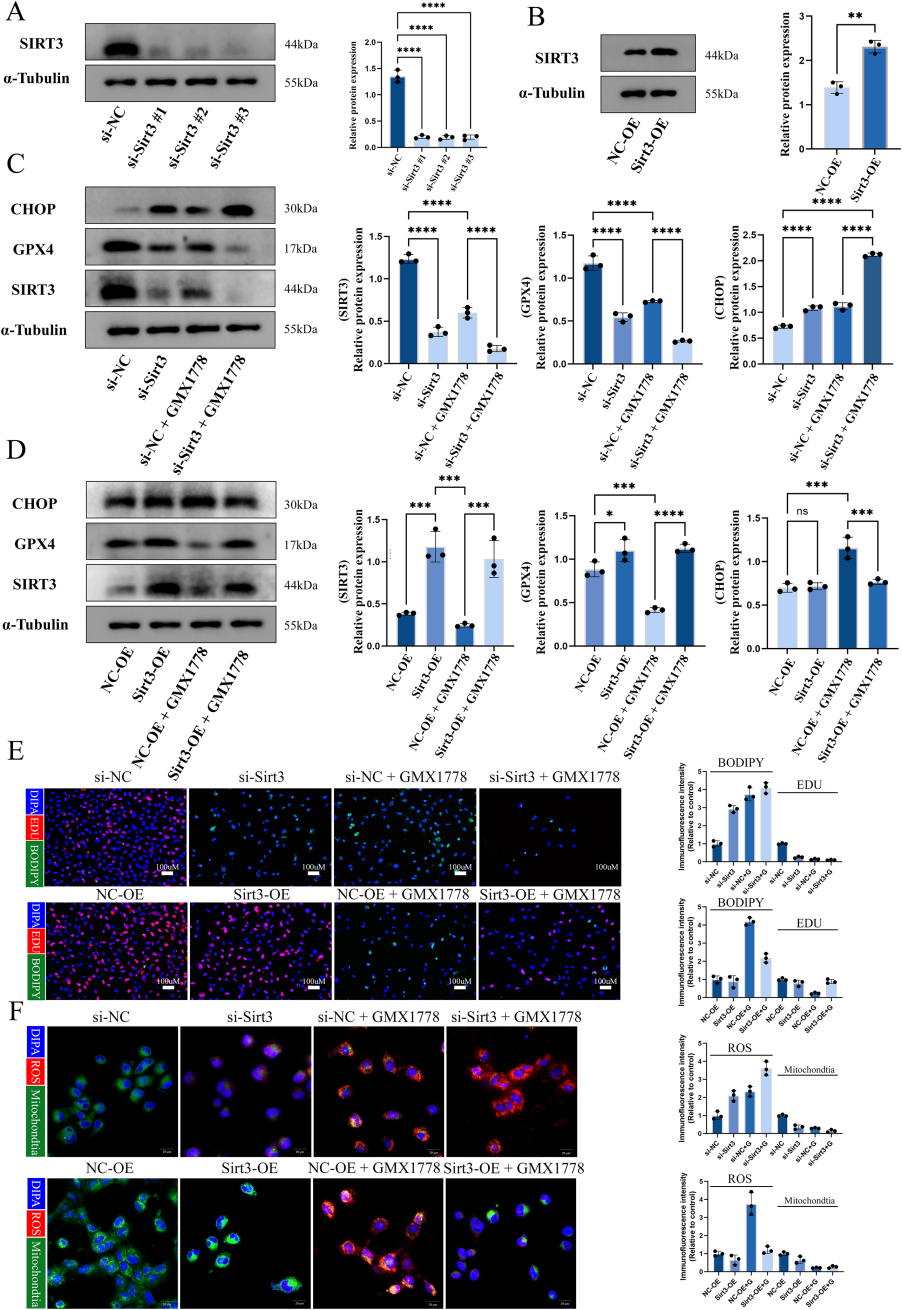
NAMPT inhibition enhances tumor cell immunogenicity via SIRT3-mediated ferroptosis. **(A)** Western blot analysis and densitometric quantification of SIRT3 protein levels after transfection with si-NC or three si-Sirt3 constructs (#1–#3). **(B)** Western blot analysis and densitometric quantification of SIRT3 protein levels after transfection with a negative control overexpression vector (NC-OE) or a Sirt3 overexpression vector (Sirt3-OE). **(C, D)** Under si-Sirt3 or Sirt3-OE conditions, cells were treated with or without GMX1778, followed by western blot analysis and quantification of CHOP, GPX4, and SIRT3 protein expression. **(E)** Immunofluorescence assays to evaluate lipid peroxidation levels and cell proliferative capacity after transfection with si-Sirt3 or Sirt3-OE. **(F)** Representative images and fluorescence intensity quantification of oxidative stress and mitochondria-associated signals assessed using an intracellular ROS fluorescent probe and mitochondrial staining. Data are representative of at least three independent experiments and are presented as mean ± SD no statistically significant difference(ns), P < 0.05 (*), P < 0.01 (**), P < 0.001 (***) and P < 0.0001 (****).

To further clarify the role of SIRT3 in NAMPT inhibitor induced ferroptosis that enhances tumour cell immunogenicity, we generated Sirt3 overexpression and knockdown constructs and transfected them into U251 cells (**Figures 4A, B**), followed by GMX1778 treatment. The results showed that, during NAMPT inhibitor–induced ferroptosis and enhanced tumour cell immunogenicity, Sirt3 knockdown exacerbated NAMPT inhibition–induced ferroptosis and endoplasmic reticulum (ER) stress, whereas Sirt3 overexpression reversed these effects (**Figures 4C, D**). In addition, immunofluorescence assays of lipid peroxidation and mitochondrial ROS further corroborated these findings. Collectively, these data indicate that SIRT3 is a critical node in NAMPT inhibitor–induced ferroptosis–triggered ER stress that enhances tumour cell immunogenicity.

### NAMPT inhibition promotes M2 to M1 polarisation of co-cultured immune cells

**Figure 5:**
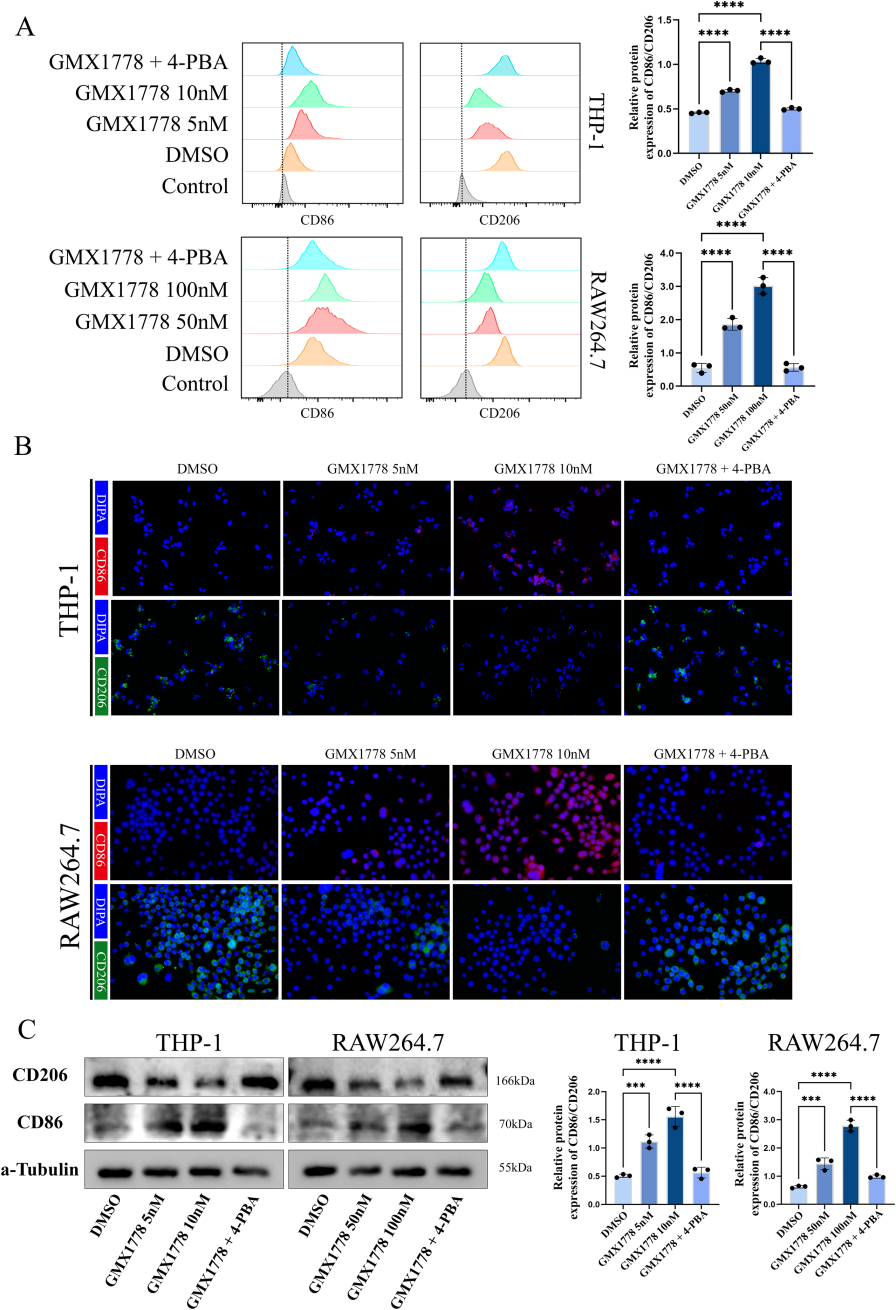
NAMPT inhibition promotes macrophage polarization from M2 to M1 phenotype. **(A)** Flow cytometry analysis showing changes in the expression of the M1 marker CD86 and the M2 marker CD206 in THP-1 and RAW264.7 cells after co-culture. **(B)** Immunofluorescence staining of CD86 (red) and CD206 (green) in co-cultured THP-1 and RAW264.7 cells; nuclei were counterstained with DAPI (blue). **(C)** Western blot analysis of CD86 and CD206 protein expression in co-cultured THP-1 and RAW264.7 cells, with corresponding quantification. Data represent at least three independent experiments and are presented as mean ± standard deviation (SD). no statistically significant difference(ns), P < 0.001 (***) and P < 0.0001 (****).

To further investigate whether NAMPT inhibition enhances tumour immunogenicity through ferroptosis-induced ER stress, we conducted a co-culture experiment as described in the Methods section. Flow cytometry analysis (**Figure 5A**) revealed that macrophages cultured in conditioned media from GMX1778-treated glioma cells exhibited a marked increase in the expression of CD86, a marker of M1 polarisation, whereas the expression of CD206, an M2 marker, was significantly reduced. These findings indicated that NAMPT inhibition enhanced tumour immunogenicity by promoting the polarisation of macrophages from an M2 phenotype to an M1 phenotype.

Notably, the addition of 4-PBA, an inhibitor of ER stress, reversed these effects, suggesting that ER stress played a critical role in mediating NAMPT inhibitor-induced immune cell polarisation. This conclusion was further supported by immunofluorescence analysis (**Figure 5B**) and Western blot results (**Figure 5C**), both of which confirmed the shift in macrophage phenotype following GMX1778 treatment.

### Anti-tumour effects of GMX1778 in a murine GBM model

**Figure 6:**
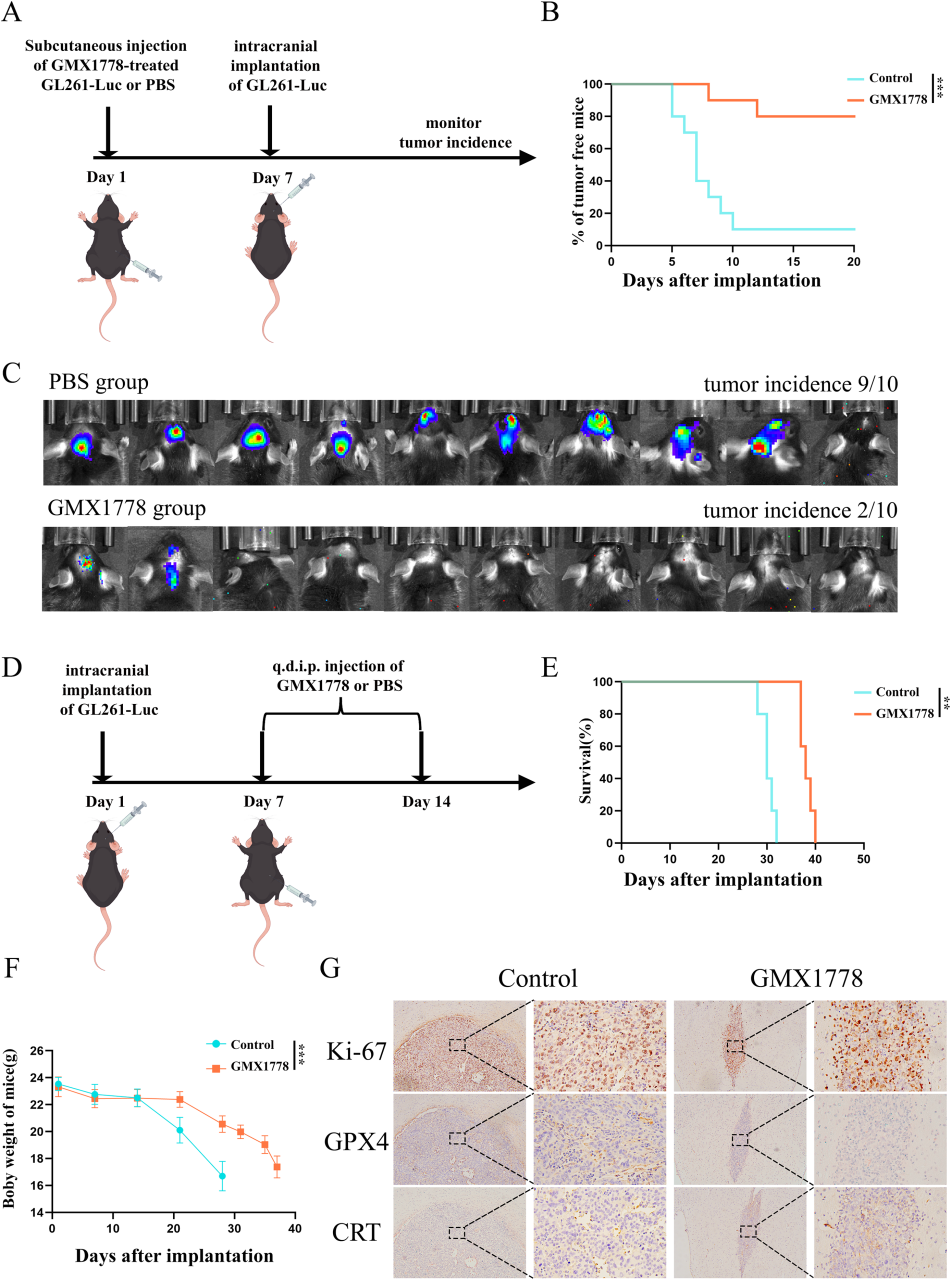
Antitumor Effects of the NAMPT Inhibitor GMX1778 in a Murine GBM Model. **(A)** In the prophylactic vaccination model, GL261-Luc cells pretreated with GMX1778 or PBS were subcutaneously injected. After 7 days, untreated GL261-Luc cells were implanted intracranially, and tumor incidence was monitored. **(B)** Tumor incidence was significantly reduced in mice pre-immunized with GMX1778-treated cells. **(C)***In vivo* bioluminescence imaging showed markedly reduced tumor signals in the GMX1778 group, with a tumor incidence of 2/10 compared to 9/10 in the control group. **(D)** Schematic of the *in vivo* treatment protocol. Following intracranial implantation of GL261-Luc cells, mice were divided into two groups and treated with GMX1778 or PBS via daily intraperitoneal injection (q.d. i.p.) for 7 consecutive days. **(E)** Kaplan–Meier survival curves showing significantly prolonged survival in the GMX1778-treated group (*p* < 0.05). **(F)** Body weight monitoring revealed a slower decline in the GMX1778-treated group compared to controls, suggesting lower systemic toxicity. **(G)** By using immunohistochemical methods, the expression changes of Ki-67, CRT and GPX in the tumor-bearing mouse section tissues were detected. Data represent at least three independent experiments and are presented as mean ± standard deviation (SD). no statistically significant difference(ns), P < 0.01 (**) and P < 0.001 (***).

To evaluate whether NAMPT inhibition enhances tumour immunogenicity, we employed a prophylactic cancer vaccination model to assess the immunogenic properties of cell death induced by GMX1778-treated glioma cells. GL261-Luc cells pre-treated with GMX1778 or PBS were subcutaneously injected into mice. After 7 days, untreated GL261-Luc cells were stereotactically implanted into the brain (**Figure 6A**). Mice pre-immunised with GMX1778-treated cells exhibited a significantly lower tumour incidence compared with the PBS group (**Figures 6B, C**), indicating that GMX1778 enhanced the immunogenic potential of tumour cell death.

To further examine the *in vivo* anti-tumour efficacy of GMX1778, we established an intracranial GBM model using GL261-Luc cells. Mice were treated with GMX1778 or PBS daily for seven consecutive days following tumour implantation (**Figure 6D**). Survival analysis demonstrated that mice in the GMX1778 group had significantly prolonged survival compared with the controls (**Figure 6E**). Additionally, the GMX1778-treated mice exhibited a markedly reduced trend in body weight loss (**Figure 6F**), suggesting a therapeutic benefit in suppressing tumour progression.

Immunohistochemical analysis further confirmed the anti-tumour effects of GMX1778 (**Figure 6G**). Ki-67 staining was used to identify tumour regions based on proliferative activity. Following GMX1778 treatment, the expression of GPX4 was significantly reduced, consistent with enhanced ferroptosis via inhibition of antioxidant defences. Moreover, the expression of CRT on the cell membrane markedly increased, reflecting enhanced immunogenicity associated with ferroptosis-induced ER stress. Collectively, these findings demonstrated that the NAMPT inhibitor GMX1778 exerted potent anti-tumour effects in GBM by promoting ferroptosis and augmenting tumour immunogenicity.

## Discussion

**Figure 7:**
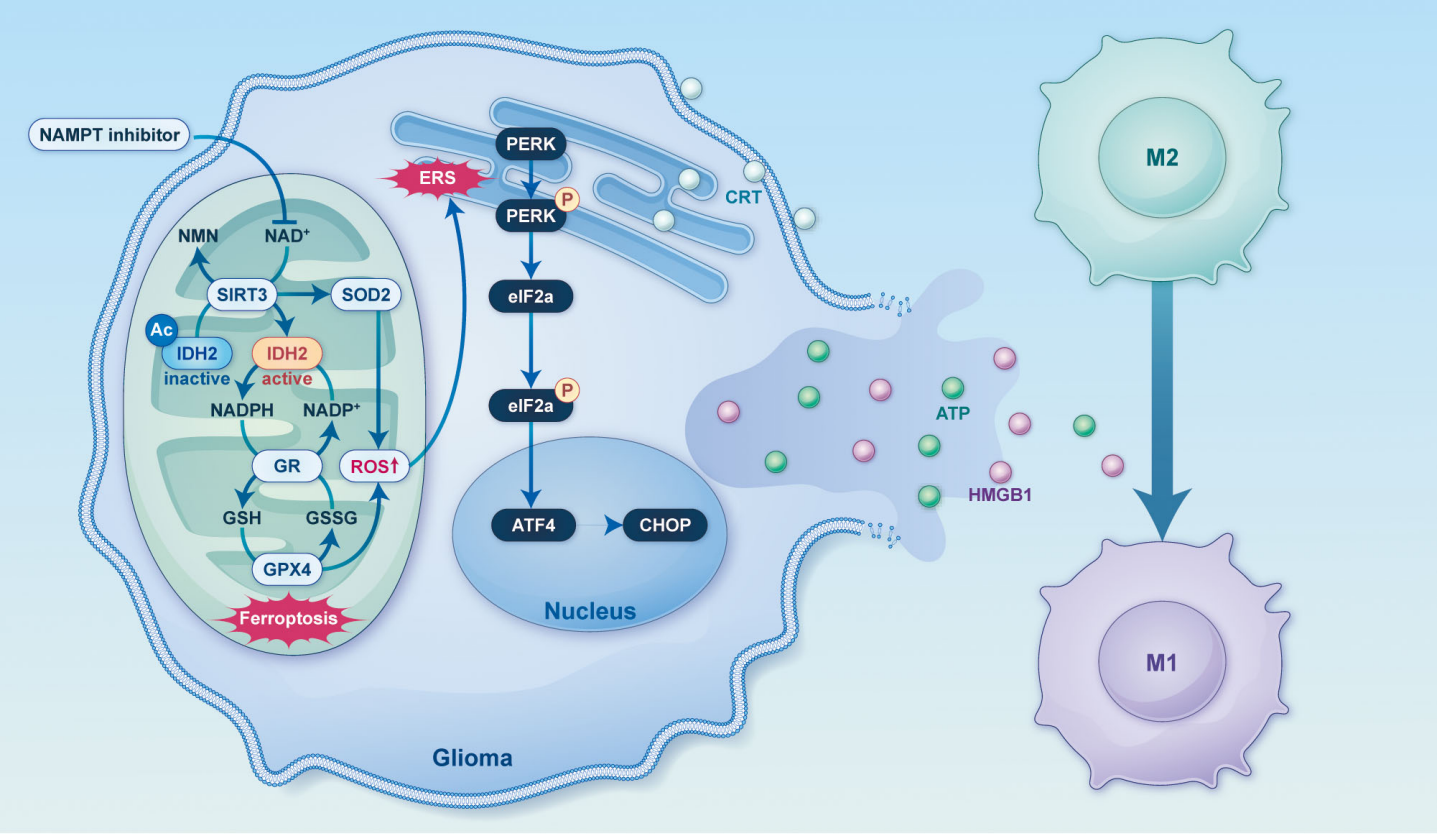
Schematic diagram illustrating the mechanism by which the NAMPT inhibitor induces ferroptosis and enhances tumour immunogenicity.

In this study, analysis of the TCGA and CGGA databases identified NAMPT as a critical target in GBM, with its high expression strongly associated with poor prognosis. Subsequent RNA sequencing of NAMPT inhibitor-treated cells revealed significant enrichment of pathways related to ferroptosis and oxidative stress. Experimental results demonstrated that the NAMPT inhibitor GMX1778 induced ferroptosis and ROS accumulation in glioma cells by down-regulating the activity of the mitochondrial NAD^+^-dependent deacetylase SIRT3. This phenomenon triggered endoplasmic reticulum stress (ERS) and led to the induction of ICD. These findings were consistent with our previous research, which showed that GMX1778 promotes specific immune alterations within the GBM tumour microenvironment in mice, characterised by the recruitment of CD3^+^, CD4^+^ and CD8^+^ T cells and a reduction in immunosuppressive M2-polarised macrophages (14). Together, these results suggest that NAMPT inhibition induces ferroptosis-mediated ERS, thereby enhancing tumour immunogenicity and offering a novel strategy to augment tumour immunotherapy (**Figure 7**).

Previous studies have shown that increasing mitochondrial NAD^+^ levels in intestinal ischemia–reperfusion injury can activate the mitochondrial deacetylase SIRT3, which activates IDH2 to promote NADPH production and enhance glutathione-mediated antioxidant defences, thereby protecting against ferroptosis (18–20). However, the role of NAD^+^ metabolic reprogramming in regulating mitochondrial SIRT3 activity and ferroptosis in glioma remains largely unexplored. In this study, through RNA sequencing and *in vitro* experiments, we demonstrated that GMX1778 reduced mitochondrial NAD^+^ levels in glioma cells, thereby down-regulating the SIRT3/IDH2/GPX4 axis, weakening mitochondrial antioxidant defences and promoting ferroptosis. Notably, the accumulation of mitochondrial ROS induced by ferroptosis not only causes oxidative damage to lipid membranes but also serves as a critical signal for the activation of intracellular stress pathways (19–22). Our findings showed that GMX1778-induced ferroptosis in glioma cells was accompanied with elevated ROS levels and robust activation of the ERS pathway, as evidenced by the up-regulation of PERK, phosphorylated eIF2α, ATF4 and CHOP. The ferroptosis inhibitor Fer-1 effectively suppressed the expression of ERS markers, whereas the ferroptosis inducer FIN56 further enhanced ERS activation, indicating that ferroptosis contributed to ER stress through ROS accumulation.

One of the key findings of this study is that NAMPT inhibition significantly enhanced tumour immunogenicity in glioma cells. Following treatment with GMX1778, we observed a marked increase in the exposure of calreticulin (CRT) on the cell surface, along with elevated levels of extracellular ATP and HMGB1, all of which are recognised as danger-associated molecular patterns (DAMPs) that serve as signals to stimulate immune responses. Given that CRT translocation is dependent on ERS (23, 24), we further investigated the role of ERS in this process. Co-treatment with GMX1778 and the ER stress inhibitor 4-PBA led to a reduction in these DAMP-associated markers, confirming that NAMPT inhibition promoted tumour immunogenicity in an ERS-dependent manner.

Previous studies have shown that CRT surface exposure and HMGB1 release play important roles in immune regulation, particularly in promoting macrophage polarisation from the M2 phenotype to the M1 phenotype, thereby enhancing anti-tumour immune responses (25). The findings of this study were consistent with these observations and further supported the notion that NAMPT inhibition enhances tumour immunogenicity through ER stress activation. Moreover, the use of a prophylactic cancer vaccination model in mice—considered the gold standard for evaluating tumour immunogenicity (21, 26–29)—provided additional evidence supporting the role of NAMPT inhibitors in enhancing tumour immunogenicity. In this model, mice pre-immunised with GMX1778-treated GL261-Luc cells exhibited a significantly lowered incidence of intracranial tumour formation upon subsequent challenge with untreated GL261-Luc cells. These results suggested that GMX1778-treated cells possess heightened immunogenicity and are capable of eliciting a robust anti-tumour immune response.

This study revealed a mechanistic link between ferroptosis and tumour immunogenicity, further emphasising the importance of ferroptosis in anti-tumour immunity. Although previous research has primarily focused on the role of ferroptosis in directly killing tumour cells (30–33), our findings demonstrated that NAMPT inhibitor-induced ferroptosis not only led to the accumulation of ROS but was also accompanied by increased release or exposure of DAMPs, including CRT translocation, ATP release and HMGB1 secretion. Notably, treatment with the ferroptosis inhibitor Fer-1 significantly reduced these effects, whereas the ferroptosis inducer FIN56 further enhanced them, indicating that ferroptosis played a critical role in shaping the immunogenic phenotype of glioma cells. This conclusion was aligned with previous reports suggesting that oxidative stress can promote immunogenicity through the enhancement of ERS (34–36). Our study supports this concept by demonstrating that ferroptosis induced by NAMPT inhibition activated the PERK–CHOP pathway, and ERS activation facilitated CRT surface exposure and HMGB1 release, thereby enhancing tumour immunogenicity.

Therefore, ferroptosis is not only a vital mechanism of tumour cell death but also serves as a regulatory driver of tumour immunogenicity and the immune microenvironment, offering a novel approach for developing ferroptosis-targeted immunotherapies. Nevertheless, this study had several limitations. Although we focused on the immunogenic effects of NAMPT inhibition, we did not evaluate its impact on other immune cell populations, such as T cells or dendritic cells. Future studies should expand the scope of immune cell profiling to comprehensively assess the multifaceted role of NAMPT inhibitors in shaping the tumour immune microenvironment.

## Data Availability

The original transcriptomic data presented in the study are publicly available. This data can be found here: Genome Sequence Archive (GSA) at the National Genomics Data Center (NGDC), accession number CRA039982 (https://ngdc.cncb.ac.cn/gsa/s/9mtEK1Kz).

## Supplementary material

Supplementary Figure 1 Effects of GMX1778 on the viability of glioma cells U251 and GL261. Cell viability was assessed using the CCK-8 assay to evaluate the effects of different concentrations of GMX1778 on U251 **(A)** and GL261 **(B)** cells at 24, 48, and 72 hours. The results showed that cell viability decreased significantly in a dose- and time-dependent manner following GMX1778 treatment.

Supplementary Figure 2 Schematic diagram of the co-culture system.Glioma cells were treated with GMX1778 for 48 hours, followed by medium replacement and an additional 24-hour incubation. The resulting conditioned medium was collected and used for macrophage co-culture. THP-1 cells were differentiated into M0 macrophages by treatment with PMA (100 ng/mL) for 24 hours. The cells were then cultured in the GMX1778-treated conditioned medium supplemented with IL-4 (50 ng/mL) and IL-13 (50 ng/mL) to induce M2 polarization. Macrophage phenotypes were assessed after 48 hours of co-culture.

Supplementary Figure 3 NAMPT expression and its prognostic value in gliomas based on the CGGA database. **(A)** Analysis of NAMPT mRNA expression levels across different WHO grades of glioma using the CGGA database. NAMPT expression was significantly higher in grade 4 gliomas (GBM) compared to grades 2 and 3. **(B)** NAMPT expression across different histological subtypes of glioma, with the highest levels observed in GBM tissue. **(C)** Kaplan–Meier survival analysis showing that patients with high NAMPT expression had significantly shorter overall survival than those with low expression (log-rank test, *p* < 0.001).

Supplementary Figure 4 Immunofluorescence analysis of CHOP expression in U251 and GL261 cells under different treatments. **(A)** U251 and **(B)** GL261 cells were subjected to immunofluorescence staining under various treatment conditions. Nuclei were stained with DAPI (blue), and CHOP, an ER stress marker, was stained in red. Merged images show co-localization of nuclear and CHOP signals. Experimental groups included: DMSO control, GMX1778-treated (10 nM for U251, 100 nM for GL261), ferroptosis inhibitor (Fer-1), GMX1778 + Fer-1, ferroptosis inducer (FIN56), GMX1778 + FIN56, and GMX1778 + ER stress inhibitor (4-PBA). GMX1778 treatment markedly increased CHOP expression. This effect was partially reversed by Fer-1, further enhanced by FIN56, and effectively suppressed by 4-PBA.

Supplementary Figure 5 Immunofluorescence Analysis of HMGB1 Release in U251 and GL261 Cells Following GMX1778 Treatment. **(A)** U251 and **(B)** GL261 cells were subjected to immunofluorescence staining under different treatment conditions. Nuclei were stained with DAPI (blue), and high mobility group box 1 (HMGB1) was labeled in red. Merged images show the overlay of nuclear and HMGB1 signals. GMX1778 treatment markedly enhanced the extracellular release of HMGB1. This effect was partially suppressed by the ferroptosis inhibitor Ferrostatin-1 (Fer-1), further enhanced by the ferroptosis inducer FIN56, and reversed by the ER stress inhibitor 4-phenylbutyric acid (4-PBA). These findings suggest that GMX1778 promotes HMGB1 release through ferroptosis and ER stress pathways, thereby enhancing tumour immunogenicity.
