# Supplementary material for: NAMPT inhibition induces ferroptosis via mitochondrial metabolic reprogramming to enhance tumour immunogenicity in glioblastoma
Source: Front Immunol. 2026 Mar 31;17:1721125. doi: 10.3389/fimmu.2026.1721125 (PMC13076486; doi:10.3389/fimmu.2026.1721125)
Supplement: Supplementary Figure 1 — Effects of GMX1778 on the viability of glioma cells U251 and GL261. Cell viability was assessed using the CCK-8 assay to evaluate the effects of different concentrations of GMX1778 on U251 (A) and GL261 (B) cells at 24, 48, and 72 hours. The results showed that cell viability decreased significantly in a dose- and time-dependent manner following GMX1778 treatment. [file DataSheet2.pdf]

Supplementary Figure 1: Effects of GMX1778 on the Viability of Glioma Cells U251 and GL261

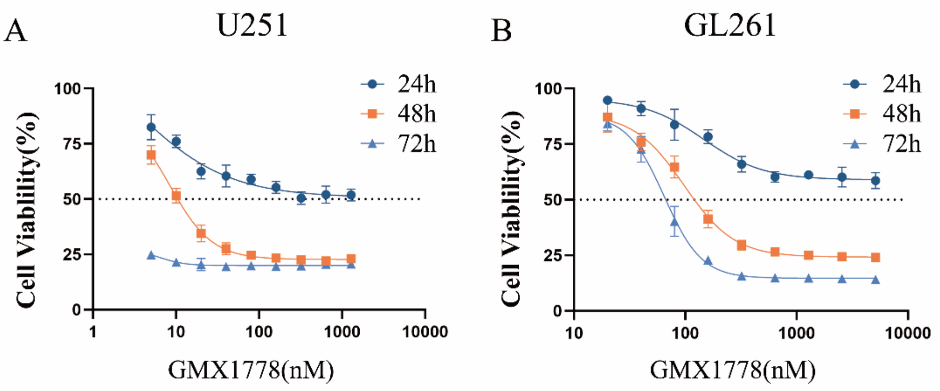


Cell viability was assessed using the CCK-8 assay to evaluate the effects of different concentrations of GMX1778 on U251 (A) and GL261 (B) cells at 24, 48, and 72 hours. The results showed that cell viability decreased significantly in a dose- and time-dependent manner following GMX1778 treatment.

Supplementary Figure 2：Schematic Diagram of the Co-culture System

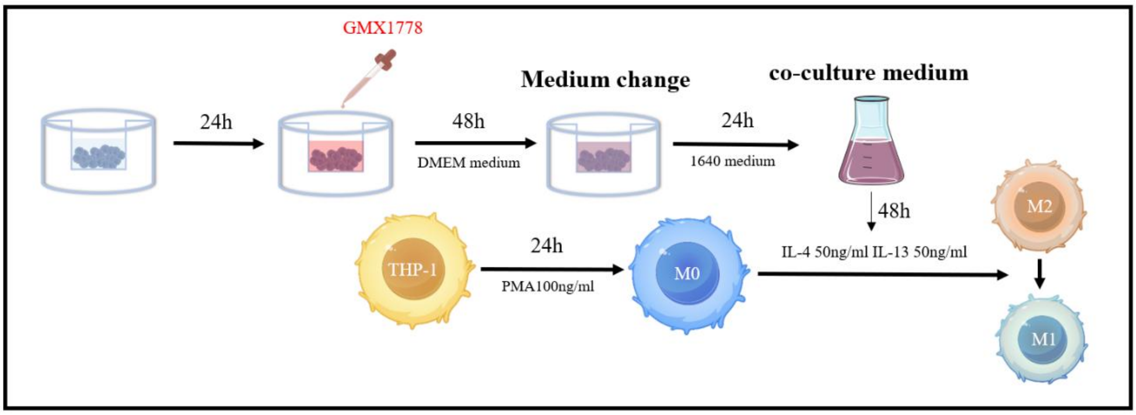


Glioma cells were treated with GMX1778 for 48 hours, followed by medium replacement and an additional 24-hour incubation. The resulting conditioned medium was collected and used for macrophage co-culture. THP-1 cells were differentiated into M0 macrophages by treatment with PMA (100 ng/mL) for 24 hours. The cells were then cultured in the GMX1778-treated conditioned medium supplemented with IL-4 (50 ng/mL) and IL-13 (50 ng/mL) to induce M2 polarization. Macrophage phenotypes were assessed after 48 hours of co-culture.

Supplementary Figure 3: NAMPT Expression and Its Prognostic Value in Gliomas Based on the CGGA Database

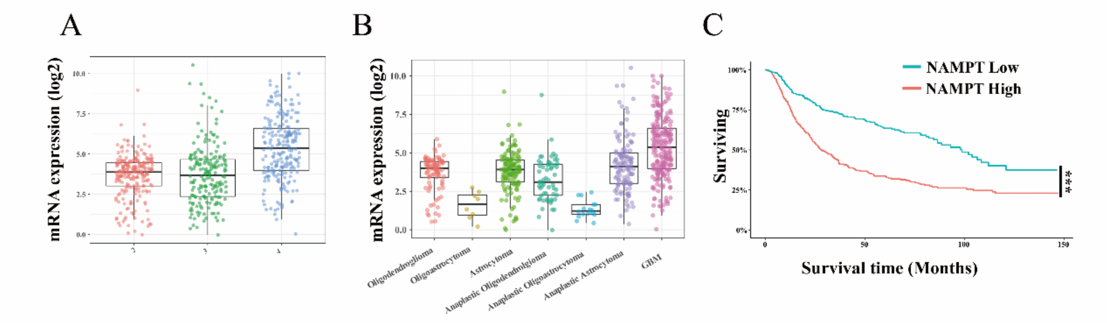


(A) Analysis of NAMPT mRNA expression levels across different WHO grades of glioma using the CGGA database. NAMPT expression was significantly higher in grade 4 gliomas (GBM) compared to grades 2 and 3.

(B) NAMPT expression across different histological subtypes of glioma, with the highest levels observed in GBM tissue.

(C) Kaplan–Meier survival analysis showing that patients with high NAMPT expression had significantly shorter overall survival than those with low expression (log-rank test, $\boldsymbol{p} < 0.001$).

Supplementary Figure 4：Immunofluorescence Analysis of CHOP Expression in U251 and GL261 Cells Under Different Treatments

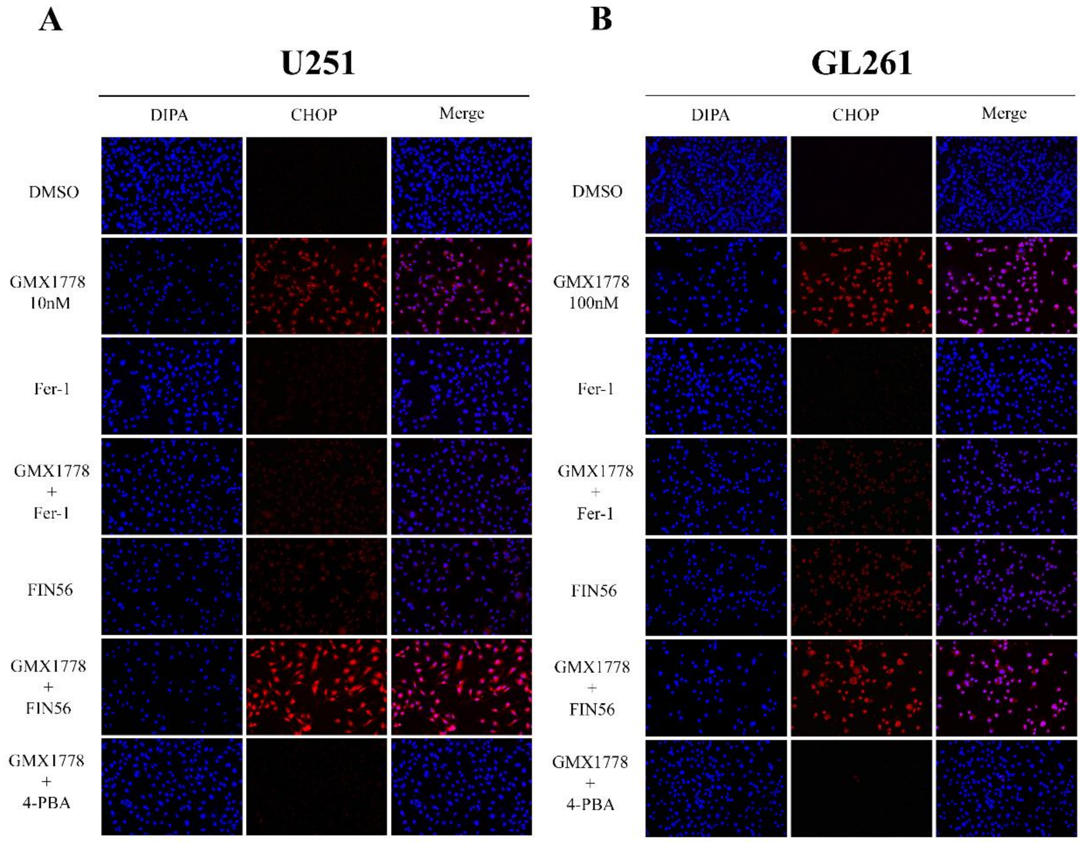


(A) U251 and (B) GL261 cells were subjected to immunofluorescence staining under various treatment conditions. Nuclei were stained with DAPI (blue), and CHOP, an ER stress marker, was stained in red. Merged images show co-localization of nuclear and CHOP signals. Experimental groups included: DMSO control, GMX1778-treated (10 nM for U251, 100 nM for GL261), ferroptosis inhibitor (Fer-1), GMX1778 + Fer-1, ferroptosis inducer (FIN56), GMX1778 + FIN56, and GMX1778 + ER stress inhibitor (4-PBA). GMX1778 treatment markedly increased CHOP expression. This effect was partially reversed by Fer-1, further enhanced by FIN56, and effectively suppressed by 4-PBA.

Supplementary Figure 5：Immunofluorescence Analysis of HMGB1 Release in U251 and GL261 Cells Following GMX1778 Treatment

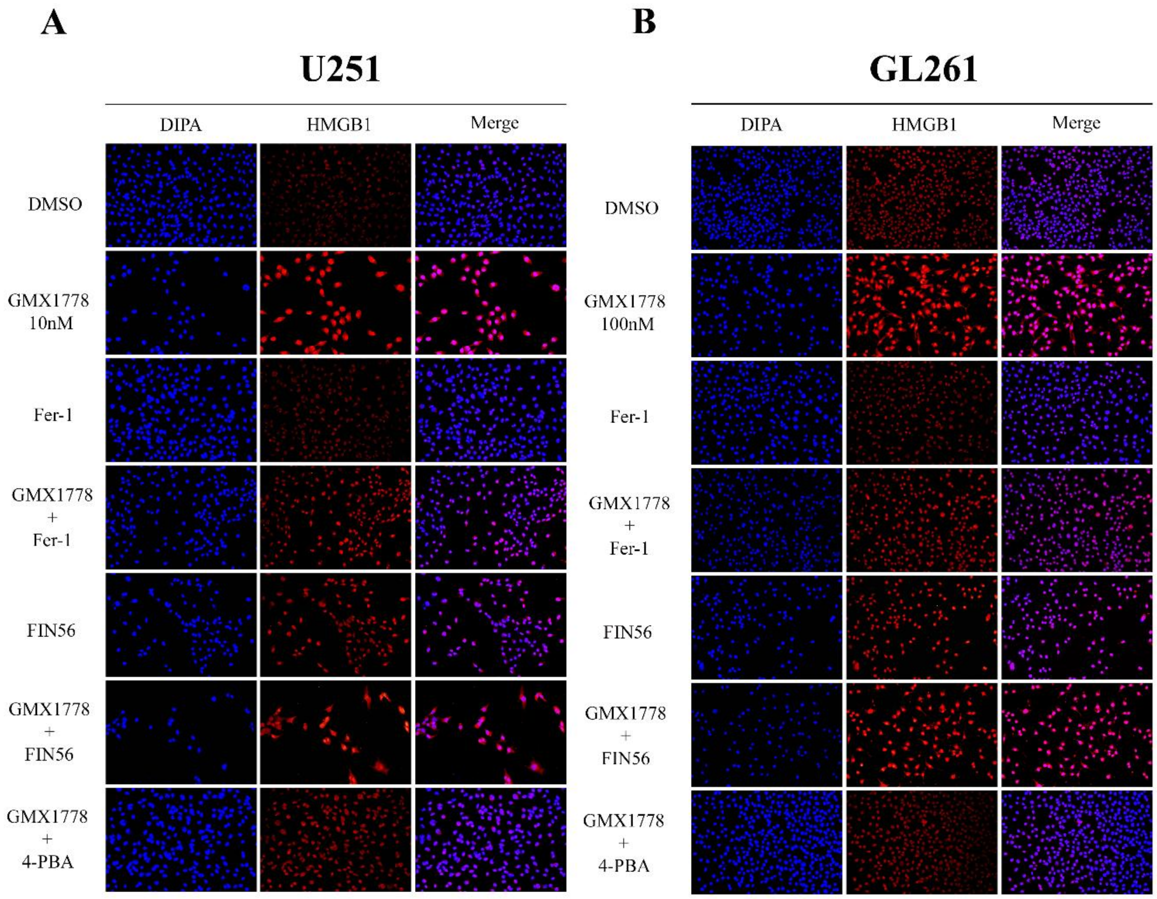


(A) U251 and (B) GL261 cells were subjected to immunofluorescence staining under different treatment conditions. Nuclei were stained with DAPI (blue), and high mobility group box 1 (HMGB1) was labeled in red. Merged images show the overlay of nuclear and HMGB1 signals.GMX1778 treatment markedly enhanced the extracellular release of HMGB1. This effect was partially suppressed by the ferroptosis inhibitor Ferrostatin-1 (Fer-1), further enhanced by the ferroptosis inducer FIN56, and reversed by the ER stress inhibitor 4-phenylbutyric acid (4-PBA).These findings suggest that GMX1778 promotes HMGB1 release through ferroptosis and ER stress pathways, contributing to the induction of immunogenic cell death (ICD).
